# Genomic, Transcriptomic, and Functional Alterations in DNA Damage Response Pathways as Putative Biomarkers of Chemotherapy Response in Ovarian Cancer

**DOI:** 10.3390/cancers13061420

**Published:** 2021-03-20

**Authors:** Sweta Sharma Saha, Lucy Gentles, Alice Bradbury, Dominik Brecht, Rebecca Robinson, Rachel O’Donnell, Nicola J. Curtin, Yvette Drew

**Affiliations:** 1 Newcastle University Centre for Cancer, Translational and Clinical Research Institute, Faculty of Medical Sciences, Newcastle University, Newcastle upon Tyne NE2 4HH, UK; sweta.sharma-saha@newcastle.ac.uk (S.S.S.); lucy.gentles@newcellsbiotech.co.uk (L.G.); a.bradbury@newcastle.ac.uk (A.B.);; 2 Department of Chemsitry/Biology, University of Konstanz, 78464 Konstanz, Germany; dominik.brecht@uni-konstanz.de; 3 Department of Biomedical, Nutritional and Sports Science, Newcastle University, Newcastle upon Tyne NE1 7RU, UK; r.robinson@newcastle.ac.uk; 4 Northern Cancer Alliance, Northern Centre for Gynaecological Surgery, Newcastle Hospitals NHS Foundation Trust, Newcastle upon Tyne NE1 4LP, UK; 5 Northern Centre for Cancer Care (NCCC), Newcastle Hospitals NHS Foundation Trust, Newcastle upon Tyne NE7 7DN, UK

**Keywords:** DNA damage repair, chemotherapy, ovarian cancer, biomarker

## Abstract

**Simple Summary:**

Several chemotherapy drugs are approved for ovarian cancer treatment in the neo-adjuvant/adjuvant setting as well as following relapse. These include carboplatin, paclitaxel, doxorubicin, topotecan, PARP inhibitors (PARPi), and gemcitabine. However, except for PAPRi, there are no predictive biomarkers to guide the choice of drug. The majority of chemotherapeutic drugs function by inducing DNA damage or inhibiting its repair. However, the association of DNA damage repair (DDR) pathway alterations with therapy response remain unclear. In this study, using a panel of 14 ovarian cancer cell lines, 10 patient ascites-derived primary cultures and bioinformatic analysis of The Cancer Genome Atlas (TCGA) ovarian cancer dataset, we identified the role of genomic/transcriptomic and/or functional alterations in DDR pathways as determinants of therapy response.

**Abstract:**

Defective DNA damage response (DDR) pathways are enabling characteristics of cancers that not only can be exploited to specifically target cancer cells but also can predict chemotherapy response. Defective Homologous Recombination Repair (HRR) function, e.g., due to BRCA1/2 loss, is a determinant of response to platinum agents and PARP inhibitors in ovarian cancers. Most chemotherapies function by either inducing DNA damage or impacting on its repair but are generally used in the clinic unselectively. The significance of HRR and other DDR pathways in determining response to several other chemotherapy drugs is not well understood. In this study, the genomic, transcriptomic and functional analysis of DDR pathways in a panel of 14 ovarian cancer cell lines identified that defects in DDR pathways could determine response to several chemotherapy drugs. Carboplatin, rucaparib, and topotecan sensitivity were associated with functional loss of HRR (validated in 10 patient-derived primary cultures) and mismatch repair. Two DDR gene expression clusters correlating with treatment response were identified, with PARP10 identified as a novel marker of platinum response, which was confirmed in The Cancer Genome Atlas (TCGA) ovarian cancer cohort. Reduced non-homologous end-joining function correlated with increased sensitivity to doxorubicin, while cells with high intrinsic oxidative stress showed sensitivity to gemcitabine. In this era of personalised medicine, molecular/functional characterisation of DDR pathways could guide chemotherapy choices in the clinic allowing specific targeting of ovarian cancers.

## 1. Introduction

Ovarian cancer accounts for ~300,000 new cancer cases globally each year and is one of the leading causes of death among gynaecological cancers, with a mortality rate of ~185,000 deaths per year [1]. Combination platinum and paclitaxel chemotherapy in conjunction with cytoreductive surgery remain the cornerstone of first-line treatment for ovarian cancers, the majority of which present at an advanced stage. Most ovarian cancers are of epithelial origin (~90%), with high grade serous ovarian cancer (HGSOC) being the most prevalent histopathological subtype [2]. Whilst the majority of HGSOCs respond to platinum-based therapy with an initial response rate of ~60%, non-HGSOC subtypes (clear cell, mucinous and low grade serous) are less responsive [3,4]. Even among women with HGSOC, the five-year overall survival remains poor (~45%), with the majority of women experiencing disease recurrence and ultimately the development of platinum resistance [5].

The disproportionate mortality rate in ovarian cancer continues to attract attention with the ambition to improve survival through the stratified provision of targeted therapies. The focus on exploiting defective DNA damage response (DDR) pathways has increased following the successful implementation of poly (ADP ribose) polymerase inhibitor (PARPi) therapy [6,7]. There is a growing armoury of cytotoxic agents and targeted therapies for ovarian cancer in both the frontline and relapsed settings. However, choice of second-line therapy for recurrent disease is determined by response and tolerability to the first-line platinum-based chemotherapy, which, in turn, is dependent on the accompanying maintenance therapies like bevacizumab and PARPis [3]. At relapse, platinum drugs may be given as single agents or as platinum doublets (combination with paclitaxel, gemcitabine ± bevacizumab, or liposomal doxorubicin) [8]. Non-platinum chemotherapy approved for second and third-line treatments and used in the resistant settings includes paclitaxel, liposomal doxorubicin, topotecan given alone or in combination with bevacizumab [3,9]. However, with the exception of stratification for PARPi therapy using BRCA mutation status, treatment strategies are not guided by molecular or functional features of individual cancers, making the prediction of response, and the selection of an appropriate agent, challenging.

Multiple studies have clearly demonstrated mutations in genes encoding proteins involved in the homologous recombination repair (HRR) pathway, e.g., BRCA, as determinants of response to both platinum agents and PARPis [10,11]. However, the understanding of the predictive nature of the DDR is not well established for the other commonly used chemotherapies in ovarian cancer. Except for taxanes, the majority of the chemotherapies approved for ovarian cancer, including carboplatin, doxorubicin, topotecan, and gemcitabine, cause cytotoxicity by inducing DNA damage, while PARPis inhibit DNA damage repair, thus increasing dependence on the DDR. Thus, it could be speculated that defects/alterations in DDR pathways at the genomic and/or functional level could serve as determinants of chemotherapy response.

This study, therefore, aimed to explore the sensitivity of five chemotherapy drugs and one PARPi used to treat ovarian cancer: carboplatin, doxorubicin, gemcitabine, paclitaxel, topotecan, and rucaparib in a panel of 14 ovarian cancer cell lines and ascites-derived primary cultures from women with ovarian cancer and their association with the genomic, transcriptomic and functional alterations of DDR genes/pathways. We identified that HRR status was significantly associated with carboplatin and rucaparib response in agreement with previous studies [12,13]. NHEJ activity correlated with better survival following doxorubicin treatment, and cell lines with high oxidative stress were found to be more sensitive to gemcitabine. Interestingly, cell line and TCGA ovarian cancer data analysis identified genomic amplification and/or high PARP10 expression to be associated with platinum-based drug response, which needs further exploration. Overall, our study identified a strong association between DDR alterations and chemotherapy response which has strong clinical implications with further validatory studies.

## 2. Materials and Methods

### 2.1. Cell line and Culture Conditions

A panel of 14 ovarian cancer cell lines was used to screen for sensitivities to chemotherapy and DDR pathway functional analysis. The clinical and morphological features of the cell lines are listed in Table S1. The mutation and copy number alteration status of the cell lines for some frequent genomic alterations commonly observed in HGSOC are listed in Table S2. Cells were incubated at 37 °C in a humidified atmosphere containing 5% CO2. They were maintained at low passage and confirmed to be mycoplasma free. All cell lines were cultured from authenticated stocks originating from ATCC or our laboratory (NUCOLL43) held in the cell line storage bank at Newcastle University.

### 2.2. Ascites Primary Cultures

Ethical approval for the collection of clinical material and data was granted (REC: 17/NE/0361), and written consent was taken. Malignant ascitic fluid was collected from patients undergoing cytoreductive surgery or symptomatic drainage of ascites, as previously described [14]. Ascites was mixed 1:1 (*v/v*) with RPMI-1640 media supplemented with 20% fetal calf serum and 1% penicillin/streptomycin. Cultures were incubated at 37 °C, 5% CO2, 95% humidified air. The medium was replenished every 4–7 days until cultures reached 70–80% confluence. Cells were then passaged for continuous culture. All experiments were carried out at early passage (<4).

### 2.3. Chemicals and Reagents

Carboplatin, gemcitabine hydrochloride, doxorubicin hydrochloride, topotecan hydrochloride hydrate, and paclitaxel were supplied by Sigma Aldrich, UK. Rucaparib was a kind gift from Pfizer Global R&D (La Jolla, CA, USA). Carboplatin, gemcitabine hydrochloride and doxorubicin hydrochloride were dissolved in 0.9% saline and topotecan, paclitaxel, and rucaparib were dissolved in Dimethyl Sulfoxide (DMSO).

### 2.4. Colony Formation Assay

Cells were plated at low density ranging from 50 to 4000 cells/well based on the plating efficiency (colonies formed/cells seeded × 100) of the cell lines and anticipated cytotoxicity of the drugs to get countable colonies (20–150) and allowed to adhere for 24 h. This was followed by treatment with different concentrations of drugs as a single agent for 24 h. The DMSO concentration was maintained at 0.5% for drugs dissolved in DMSO. Following 24 h of drug treatment, the medium was replaced with a drug-free medium and cells incubated until colonies >30 cells formed. Cells were fixed in methanol: acetic acid (3:1 *v/v*) and stained with 0.4% crystal violet. Colonies were counted, and the % survival for each treatment calculated from the relative plating efficiency of treated versus vehicle (0.5% DMSO/only media) controls. The LC_50_ values were calculated from individual experiments where the concentration causing a 50% decrease in cell survival was calculated by interpolation of the survival curves. The % Survival was also estimated from the individual survival curves at a selected concentration. The mean and standard deviation of the LC_50_ and % Survival values were calculated further from 3 independent experiments.

### 2.5. Sulforhodamine B Assay (SRB) 

Primary cultures were seeded into 96-well plates (1000 cells/100 μL per well) and allowed to adhere overnight. Media was replaced with fresh medium containing increasing concentrations of each drug which was again replaced with fresh medium after 24 h and incubated for a further 5–6 days or until the control wells were subconfluent. Cells were fixed with 50% (*w/v*) trichloroacetic and stained with 0.4% SRB. Absorbance was measured at 570 nm using a BMG FLUOstar Omega plate reader, and the growth as a percentage of vehicle control was calculated (% survival) for each drug concentration.

### 2.6. Databases and Tools

Cancer Cell Line Encyclopedia (CCLE) and The Cancer Genome Atlas (TCGA) data was accessed using cbioportal (https://www.cbioportal.org/) (accessed on 12 January 2021).

### 2.7. Bioinformatics Analysis

RNA-Seq data was available from the CCLE database for 9/14 cell lines. The normalised read counts were correlated with drug sensitivity to generate the Pearson’s correlation coefficient, which was used to perform hierarchical clustering. Additionally, exome sequencing data was used to identify key genetic alterations, including copy number alterations and mutations in the cell line panel. Additionally, RNA-Sequencing and genomic data, including mutation and copy number alterations, were downloaded for 201 ovarian cancer patients included in the TCGA pan-cancer Atlas study using cbioportal. Published in house RNA-Seq data on 2567 genes generated using Illumina NextSeq with the HTG EdgeSeq Oncology biomarker panel assay (Gene Expression Omnibus submission: GSE150942) (13) was used for validation of the correlation of gene expression with chemotherapy response.

### 2.8. HRR Function by Immunofluorescence Based γH2AX-RAD51 Assay

HRR was assessed by immunofluorescence microscopy, as previously described [15]. Cells were treated for 48 h with 10 μM rucaparib alongside vehicle alone controls (0.5% DMSO). To quantify DNA damage, cells were stained with mouse monoclonal anti-phospho-histone H2A.X (Ser139) antibody (Upstate/Millipore, Burlington, NJ, USA) at 1:1000 and rabbit monoclonal anti-RAD51 antibody (AbCam, Cambridge, UK) at 1:500 to assess downstream HRR functional repair. Secondary antibodies used were Alexa 488 conjugated goat anti-rabbit and Alexa 546 conjugated goat-anti mouse (Invitrogen, Waltham, USA), both at 1:1000. The nuclei were stained with DAPI. γH2AX and RAD51 foci in each cell were quantified using ImageJ software and data analysed using GraphPad Prism. A >2-fold increase in γH2AX foci formation was used as an indicator of DNA damage induction following rucaparib treatment, and a >2-fold increase in RAD51 foci formation was indicative of functional HRR. The same method was also used for analysing the HRR ability of primary cultures with minor modifications.

### 2.9. Non-Homologous End Joining (NHEJ) Plasmid Re-Joining Assay

NHEJ plasmid re-joining assay was performed as described previously by Bradbury et al. [13]. Equimolar quantities of pimEJ5GFP (Addgene plasmid # 44026 that was originally a kind gift from Jeremy Stark and Maria Jasin [16], and pCBASceI (previously described) plasmids were co-transfected into the cell lines using Lipofectamine 3000 reagent according to the manufacturer’s instructions. The cells were then collected by trypsinisation, washed with PBS and resuspended in 500 µl of PBS for analysis using flow cytometry to estimate the %GFP positive cells. Fifty-thousand cells were analysed per sample to estimate the %GFP positive cells. The %NHEJ activity of the co-transfected cells was calculated by normalising with %GFP positive cells in untransfected cells. Pearson’s correlation coefficient analysis was performed to correlate %NHEJ activity of each cell line with % survival at fixed concentrations of the drugs.

### 2.10. Measurement of 8-OHdG Levels in the DNA

Cells were seeded at 0.5 × 106 cell/mL in 100 mm dishes and cultured for two doubling times before harvesting, snap freezing and storing the cell pellet at −80 °C prior to DNA isolation. The snap-frozen cell pellets were rapidly thawed at 37 °C and DNA isolated using the QIAamp^®^ DNA Mini Kit (Qiagen, Hilden, Germany) according to the manufacturer’s instructions. DNA was quantified using a NanoDrop™ ND-1000 Spectrophotometer (Agilent, Santa Clara, CA, USA). Using the HT 8-oxo-dG ELISA Kit II (R&D Systems Inc^®^, 4380-096-K, Minneapolis, MN, USA), samples were treated with DNase and alkaline phosphatase each for one hour at 37 °C as per manufacturer’s instructions. Equal quantities of DNA from each cell line were analysed for 8-OHdG levels. The absorbance (450 nm) measured following detection with horseradish peroxidase (HRP) and TACS-Sapphire™ colourimetric substrate corresponds to the antibody bound to the pre-coated 8-OHdG on the well. The absolute 8-OhdG levels were calculated using serial dilutions of the standard supplied with the kit.4.6.1 TP53 functional analysis. Pearson’s correlation coefficient analysis was performed to correlate 8-OhdG level/µg of DNA of each cell line with % survival at fixed concentrations of the drugs.

### 2.11. Statistical Analysis

Statistical comparison of datasets between two defined groups was performed using a non-parametric *t*-test. Pearson’s correlation coefficients were calculated for all correlation analyses. Hierarchical clustering of Pearson’s correlation coefficients was performed to identify drug sensitivity clusters and the associated gene expression clusters. All statistical analysis was performed using Graphpad Prism 8 and statistical packages in R. The following *p*-value cut-offs were used: * *p*-value < 0.05; ** *p*-value < 0.01; *** *p*-value < 0.001.

## 3. Results

### 3.1. Colony Formation Cell Survival Assay Demonstrates a Range of Sensitivities to the Six Systemic Therapies across the Ovarian Cancer Cell Line Panel

**Figure 1:**
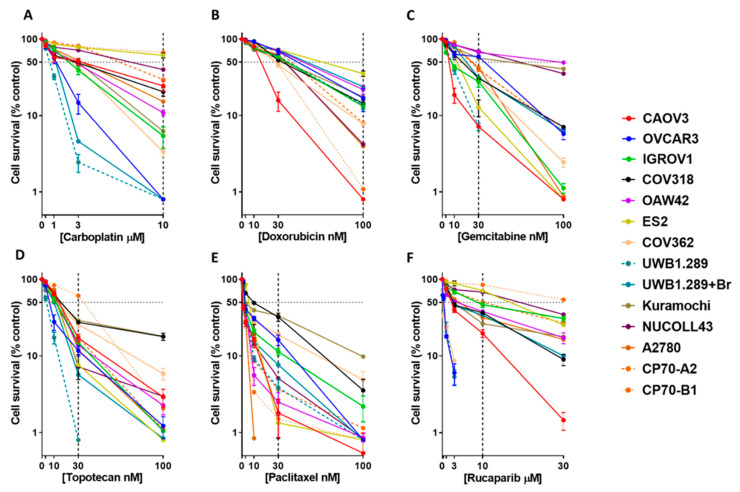
Cytotoxicity studies using a panel of 14 ovarian cancer cell lines identifies a spectrum of sensitivities for the six chemotherapeutic drugs. Colony formation assays to estimate the cytotoxic potential of (**A**) carboplatin (0–10 µM), (**B**) doxorubicin (0–100 nM), (**C**) gemcitabine (0–100 nM), (**D**) topotecan (0–100 nM), (**E**) paclitaxel (0–100 nM), and (**F**) rucaparib (0–30 µM). Each data point represents the mean and standard deviation of three biological replicates for each cell line.

The cell line panel (described in Tables S1 and S2) demonstrated a spectrum of sensitivities to the cytotoxicity of the chemotherapeutic agents with the fold-difference between the highest and lowest LC_50_ values ranging from 150-fold for rucaparib, 31-fold for carboplatin, 14-fold for gemcitabine, 11-fold for topotecan and paclitaxel, and 4-fold for doxorubicin (Table 1, Figure 1, Figure S1).

**Figure 2:**
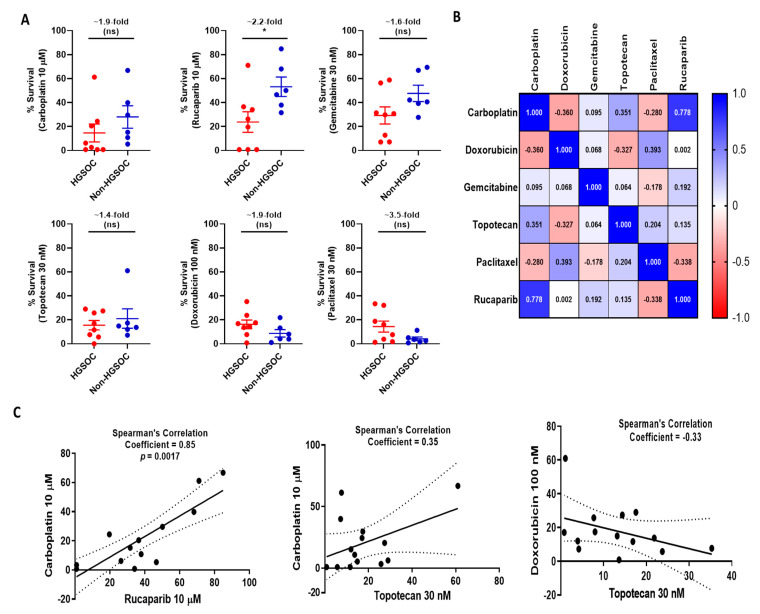
High grade serous (HGSOC) and non-high grade serous (Non-HGSOC) cell lines show distinct differences in their sensitivities to chemotherapeutic agents with different drugs correlating differently with others. (**A**) Survival of the HGSOC compared with the Non-HGSOC cell lines for carboplatin, doxorubicin, gemcitabine, topotecan, paclitaxel, and rucaparib. Plots represent the mean ± SEM of the % survival of cell lines in each group. Fold-changes are calculated between the mean of the two groups (ns: statistically non-significant; * *p* < 0.05) (**B**) Survival at fixed concentrations of drugs are shown in the heatmap, and hierarchical clustering identified a strong association between carboplatin and rucaparib with topotecan being the next closest member. Gemcitabine, doxorubicin, and paclitaxel formed a second distinct sub-cluster (**C**). Correlation analysis identified a statistically significant correlation between carboplatin and rucaparib sensitivity. A positive correlation was observed between carboplatin and topotecan, and a negative correlation was observed between doxorubicin and topotecan (although statistically non-significant).

Since the LC_50_ values were derived by interpolation from the curve and showed limited variation across the cell lines, the % survival (± SD) at a fixed drug concentration was used for further analysis (Table 1, Figure S2). The concentration selected for each drug was that which gave the greatest range of cytotoxicity between the cell lines. To assess the robustness of this metric, we also checked to see if the % survival at selected concentration correlated with the Area Under the Curve (AUC), which is another metric used by previous studies like Cancer Therapeutics Response Portal (CTRP) (https://portals.broadinstitute.org/ctrp/)(Last accessed on 12 January 2021). We observed a statistically significant positive correlation between the two parameters (Table S3). The cell lines were stratified into ‘HGSOC’ (Kuramochi, COV318, CAOV3, ES2, UWB1.289, UWB1.289 + BRCA1, NIH-OVCAR3, and COV362), and ‘non-HGSOG’ (OAW42, A2780, CP70-B1, CP70-A2, IGROV1, and NUCOLL43) based on published data [12]. The HGSOC subgroup showed greater sensitivity to carboplatin (1.9-fold), doxorubicin (1.9-fold), gemcitabine (1.6-fold), topotecan (1.4-fold), and rucaparib (2.2-fold) cytotoxicity, which was statistically significant only for rucaparib (*p* = 0.03), in comparison to the non-HGSOC subgroup. On the other hand, paclitaxel sensitivity was greater (3.5-fold) among cell lines grouped in the ‘non- HGS’ subgroup, although not statistically significant (Figure 2A).

In line with clinical observations [11], a strong positive correlation was observed between sensitivities (% survival) to the platinum agent, carboplatin, and the PARPi, rucaparib (Pearson’s correlation co-efficient = 0.85; *p*-value = <0.001). UWB1.289, COV62, and NIH-OVCAR3 were the most sensitive to both drugs. NIH-OVCAR3 data was previously published by Bradbury et al. [13] and was included here for the completeness of the analysis. A positive correlation was also observed between carboplatin and topotecan sensitivity (Pearson’s correlation co-efficient = 0.44; *p*-value = 0.11). Conversely, a negative correlation was seen between doxorubicin and topotecan sensitivity (Pearson’s correlation co-efficient = −0.4; *p*-value = 0.15) (Figure 2B,C, Figure S3). No other drug pairs correlated (Figure 2C).

### 3.2. Key Genetic Alterations Associated with Response to Chemotherapies.

**Figure 3:**
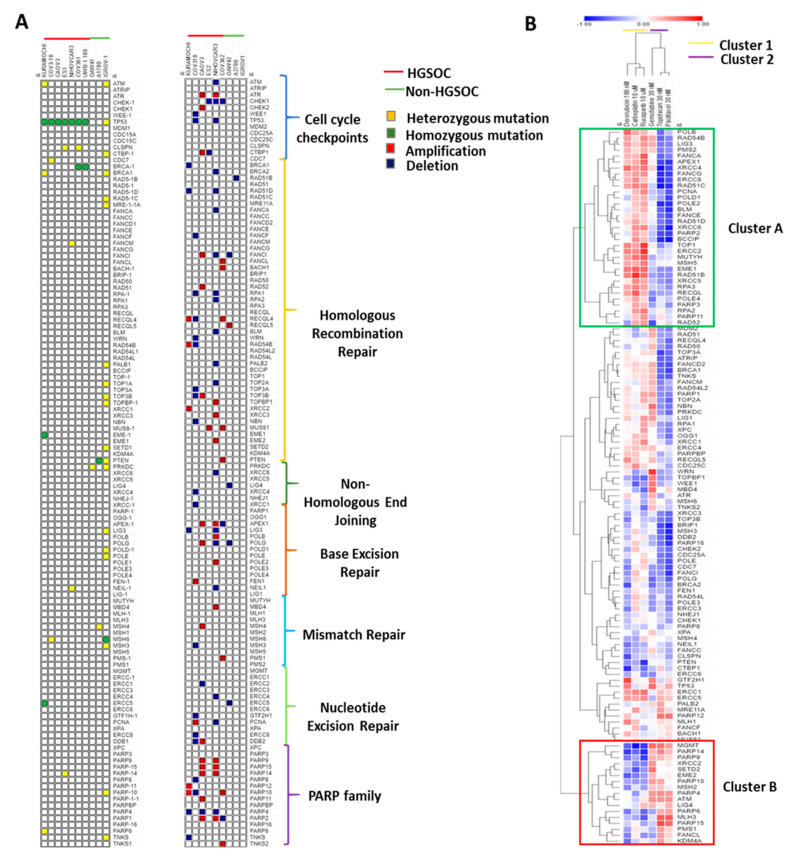
Genomic and transcriptomic alterations in the DNA damage repair (DDR) pathway determine response to chemotherapy in ovarian cancer cells (**A**) Mutation and copy number alteration profile of 120 DDR-regulatory genes among HGS and Non-HGSOC cell lines and the corresponding DDR pathways, (**B**) Hierarchical clustering of the Pearson’s correlation coefficient obtained by correlating gene expression values with the % survival of the cell lines at fixed concentrations of the drugs (shown in the figure). (Genome sequence data were available for 10/14 cell lines in the panel. Copy Number Analysis and RNA-Seq data for UWB1.289 cell line were not available).

The wide spectrum of drug sensitivities across the cell line panel led us to investigate differences in the genomic landscape of DDR pathways as determinants of drug sensitivity. Firstly, the cell line profiles were confirmed by correlating the published CCLE RNA-Seq data with the targeted RNA-Seq data (in-house) for 2495 genes highlighting a positive correlation across most (91%) of the genes (Figure S4), confirming the cell line authenticity. With the focus of this study being DDR pathways, we explored the genomic status (mutation and copy number alteration) of 120 genes that are either involved in cell cycle checkpoint or DNA damage repair pathways (HRR, NHEJ, BER, MMR, NER, and PARP family proteins) among the 10 cell lines included in our panel (data was not available for four cell lines). As typical of ovarian cancers, genomic analysis of the cell line panel confirmed that the number of copy number alterations (CNAs) (Average no. of CNAs = 2112) was much higher than mutations (Average no. of mutations = 666). A greater frequency of mutation was observed among the non-HGSOC cell lines (average no. of mutations = 1408) than the HGSOC cell lines (average no. of mutations = 349), with the IGROV1 cell line having the highest number of mutations (3227), and the NIH-OVCAR3 and UWB1.289 cell lines having the lowest (225). Analysis of the mutation status of the 120 DDR genes identified that homozygous loss of TP53 was exclusive to HGSOC cell lines, and most mutations in the DDR genes among the cell lines were heterozygous and unlikely to impact on function. The few homozygous mutations were seen in BRCA1 in the COV362 and UWB1.289 cell lines, ERCC5 and EME1 in the Kuramochi cell line, PTEN in the A2780 cell line, and MSH6 in the IGROV1 cell line, highlighting the likely role of these genes in impairing the associated pathway and hence determining chemotherapy response (Figure 3A). As expected, only BRCA1 loss could be associated with increased sensitivity of the cell lines COV362 and UWB1.289 to carboplatin and rucaparib (Figure 1A,F).

### 3.3. DDR Gene Expression Correlates with Sensitivity to the Different Chemotherapy Drugs in Cell Lines.

While CNA status did not show any strong association with chemotherapy response, the differences in mRNA levels were more likely to show variation across cell lines. Therefore, to understand the significance of differential mRNA expression of the 120 DDR genes with drug response, we correlated the log-transformed RSEM values as a measure of gene expression derived from the database with the % survival for each drug at a fixed concentration (Table 1). Hierarchical clustering of the Pearson’s correlation coefficients identified two distinct drug clusters: Cluster1 included carboplatin, doxorubicin, and rucaparib, and Cluster 2 included gemcitabine, topotecan, and paclitaxel. Hierarchical clustering also identified two gene clusters: Cluster A (33 genes) and Cluster B (17 genes), correlating with dramatically contrasting drug sensitivity profile. For genes in Cluster A, expression levels correlated positively with % survival with carboplatin, doxorubicin, and rucaparib treatment and hence likely associated with treatment resistance and vice versa for paclitaxel and topotecan. The reverse pattern was true for Cluster B, where the expression profile of genes in Cluster B correlated positively with paclitaxel and topotecan sensitivity and negatively to the rest of the drugs. While Cluster A was dominated by genes belonging to the HRR (RAD51 proteins, RPAs, FANC family proteins, etc), NHEJ (XRCC4, XRCC5 and XRCC6), and BER pathways (POLE, PCNA, etc), Cluster B predominantly included genes belonging to the PARP family members (PARP14, PARP9, PARP10, PARP4, PARP6, and PARP15) (Figure 3B). The gene expression correlations were further validated using in-house RNASeq data for 37 out 50 genes (available in the panel of 2567 genes) belonging to Clusters A and B. Hierarchical clustering of the correlation coefficients using this dataset also identified a similar clustering pattern, thus validating the findings (Figure S5).

### 3.4. DDR Gene Expression Correlates with Sensitivity to Platinum Chemotherapy in HGSOCs in the TCGA Cohort.

**Figure 4:**
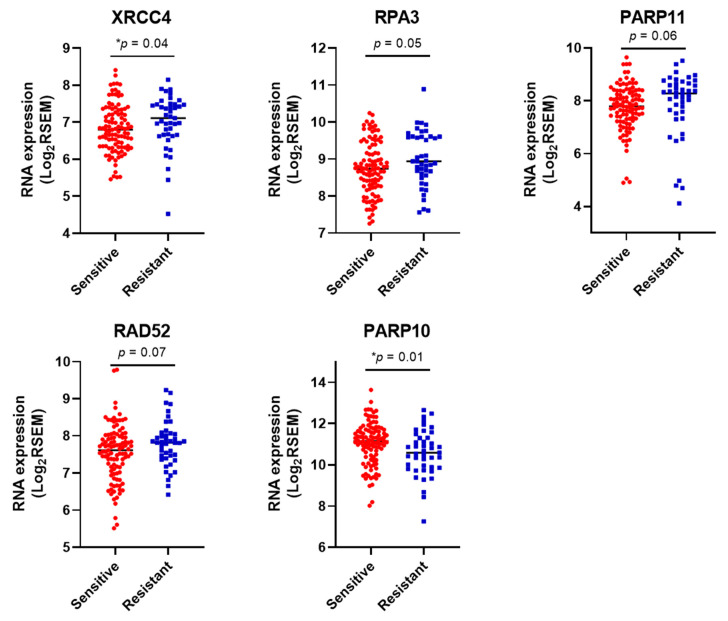
Differential expression of five out of 50 DDR genes between Platinum-sensitive and resistant ovarian cancers in the The Cancer Genome Atlas (TCGA) dataset (*n* = 148). Fifty genes correlating with chemotherapy response identified through the clustering analysis were analysed for differential expression between the Platinum sensitive (*n* = 105) and resistant (*n* = 43) sub-groups of ovarian cancers in the TCGA dataset. The expression profile of five genes most differentially expressed between the two sub-groups is shown here. The log2RSEM values for each gene are plotted on the *y*-axis (* *p* < 0.05). Data for RAD51B and RAD51D unavailable.

To confirm the likely association, we further analysed the data of 201 HGSOC ovarian cancers (using TCGA data) with platinum response status for 190 patients. We tested for any differential expression of 48 genes that formed the Clusters A and B (Figure 3B) between the platinum sensitive (*n* = 105) and resistant (*n* = 43) groups of patients. While most genes followed a similar differential expression trend, as observed in the cell lines, the top five differentially expressed genes were XRCC4, RPA3, PARP11, RAD52, and PARP10 (Figure 4). PARP10 was amplified/highly expressed among 44% of ovarian cancers, of which 42% were platinum sensitive, and only 9% were resistant. This highlights a possible role for PARP10 amplification/high expression as a predictor of platinum (and PARPi/doxorubicin) sensitivity among patients. Interestingly, all cancers with PARP10 amplification also showed co-occurrence of REQL4 amplification, similar to the co-occurrence profile seen in the cell lines Kuramochi and COV362.

### 3.5. Functional Status of DDR Pathways Determine Response to Chemotherapy

**Figure 5:**
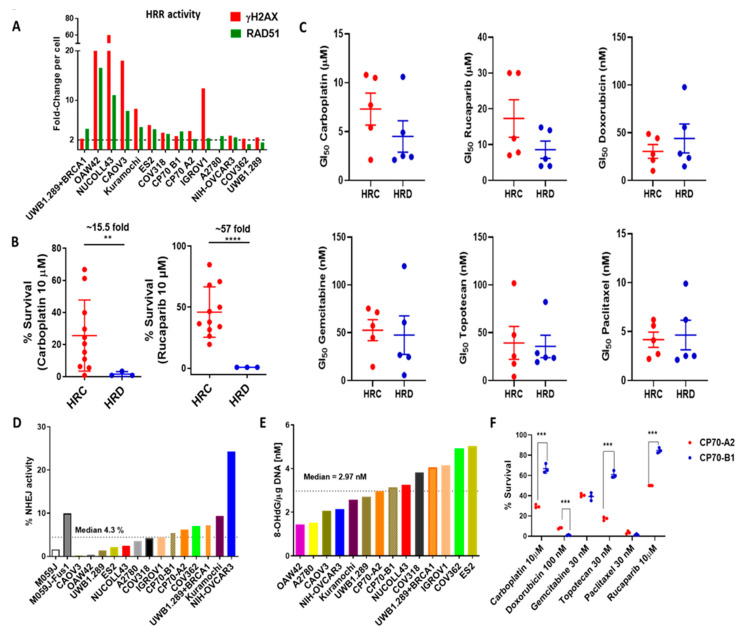
Functional/activation status of the DDR pathway and their association with chemotherapy response in cell lines. (**A**) Homologous recombination repair status of cell lines as determined by γH2AX-RAD51 foci formation assay. A >2-fold increase in RAD51 foci (green) formation is indicative of functional HRR following DNA damage induction measured by γH2AX-foci (red), identifying COV362 and UWB1.289 cell lines as HRD. Due to the pan-nuclear staining of γH2AX in the A2780 cell line, the γH2AX-foci could not be measured. Data are from a single representative experiment with a minimum count of 100 cells per cell line. (**B**) HRD cell lines were statistically significantly more sensitive to carboplatin and rucaparib. Each data point represents mean % survival at a given concentration of technical replicates of three independent experiments. (**C**) HRD primary cultures were more sensitive to carboplatin and rucaparib while HRC primary cultures were more sensitive to doxorubicin (statistically non-significant) as seen by the difference in the mean in GI_50_ values determined by Sulforhodamine B (SRB) assay, each data point represents a single patient-derived primary culture (**D**) %NHEJ activity measured by plasmid re-joining assay. Data represent the mean value of duplicate technical experiments. (**E**) Baseline 8-OHdG/µg DNA (nM) for each cell line and its correlation with % survival with each drug at a given concentration. Data represent the mean value of duplicate technical experiments. (**F**) The MMR-deficient cell line CP70-A2 was significantly more sensitive to carboplatin, topotecan, and rucaparib and less sensitive to doxorubicin than CP70-B1 cell line. Each data point represents mean % survival at given concentration of technical replicates of three independent experiments (** *p*-value < 0.01; *** *p*-value < 0.001, **** *p*-value < 0.0001).

To assess the association between the functional activity of DDR pathways and response to chemotherapy, we first characterised the cells for their HRR ability using the RAD51 focus formation assay. Twelve of fourteen cell lines showed >2-fold increase in RAD51 foci formation following the induction of DNA damage and were classified as HRR competent (HRC) (Figure 5A). However, we have previously shown NIH-OVCAR3 cells to have defective HRR pathways by plasmid re-joining functional assay, despite the ability to form RAD51 foci (13) and hence were categorised as HRR deficient (HRD). The remaining two cell lines, COV362 and UWB1.289, were also classified as HRD, as expected due to the functional loss of BRCA1 in both cell lines (Figure 5A). The mean % survival of HRC cell lines was found to be significantly higher than HRD cells for both carboplatin (15.5-fold) and rucaparib (57-fold) (Figure 5B). No statistically significant difference was seen between the HRC and HRD groups with respect to sensitivities to other drugs analysed.

The association of HRR status with chemotherapy response was further validated using ascites-derived primary cultures from chemotherapy naïve HGSOCs. We analysed 10 primary cultures for their functional HRR status using the RAD51-γH2AX foci assay and the sensitivity to chemotherapy using growth inhibition assay. Fifty-percent of the samples were found to be HRD (Table S4), as has been reported previously [17,18]. Genomic analysis of BRCA mutation status identified one patient with a germline BRCA1 mutation and another with a somatic BRCA1 mutation (Table S4); all others were BRCA-wt (*n* = 5) or unknown (*n* = 3). We identified a spectrum of sensitivities across the samples with the fold-difference between the highest and lowest GI_50_ value (dose of the drug causing 50% growth inhibition) ranging from 38-fold for topotecan, 26-fold for rucaparib, 21-fold for gemcitabine, 10-fold for doxorubicin, and 5-fold for carboplatin and paclitaxel (Figure S6 and Table S4). Similar to the observations in cell lines, HRD cancers were more sensitive to carboplatin (HRC mean GI_50_ ± SEM = 7.3 ± 1.6 µM; HRD mean GI_50_ ± SEM = 4.5 ± 1.6 µM) and rucaparib (HRC mean GI_50_ ± SEM = 17.3 ± 5.2 µM; HRD mean GI_50_ ± SEM = 8.6 ± 2.4 µM). In contrast, HRC cancers were more sensitive to doxorubicin (HRC mean GI_50_ ± SEM = 30.4 ± 7.1 nM; HRD mean GI_50_ ± SEM = 43.9 ± 15 nM) (Figure 5C and Figure S6).

A plasmid re-joining assay was used to estimate the NHEJ activity of the cell line panel. A wide spectrum of %NHEJ activity was observed across the cell line panel ranging from 0.2% (CAOV3) to 24.24% (NIH-OVCAR3) with a median of 4.3%. To identify any association with chemotherapy response, the NHEJ activity of each cell line was correlated with % survival at fixed drug concentration. The distribution of NHEJ activity highlighted two cell lines, OAW42 and ES2, as outliers in terms of correlation with chemotherapy response and hence were removed. The %NHEJ activity correlated positively with response to doxorubicin (as expected), gemcitabine, and paclitaxel and correlated negatively with carboplatin and rucaparib response. Survival following topotecan exposure did not correlate with NHEJ activity (Figure 5D; Figure S7A; Table S5).

Genomic analysis identified BER pathway alterations as the next most frequent alterations after HRR. The main endogenous lesion repaired by this pathway is oxidised guanine at the eighth-position resulting from oxidative stress. Therefore, we measured the baseline oxidative stress of each cell line by quantification of 8-OHdG levels/µg of DNA as an indirect measure of intrinsic BER pathway activation. The highest baseline 8OHdG levels were observed in the ES2 cell line (5.02 nM) and the lowest in the OAW42 cell line (1.43 nM) with a median concentration of 3.05 nM. Correlation with survival showed the strongest trend towards negative correlation with gemcitabine. Gemcitabine has been reported to enhance reactive oxygen species (ROS) production and consequently oxidative stress; therefore, this observation highlighted that it is likely that gemcitabine would be more cytotoxic in cell lines with poor BER ability resulting from oxidative DNA damage. There was a weaker correlation with cytotoxicity with other drugs (Figure 5E; Figure S7B; Table S6).

To understand the likely effect of MMR loss, we compared the sensitivities between MMR-competent (CP70-B1) and MMR-defective (CP70-A2) cell line pairs for their response to all six drugs. The CP70-A2 cells were significantly more sensitive to carboplatin (2.3-fold), topotecan (3.5-fold), and rucaparib (1.7-fold); the CP70-B1 cells showed greater sensitivity to doxorubicin (7.4-fold). There was no difference in sensitivity to gemcitabine and paclitaxel between the cell line pairs (Figure 5F).

## 4. Discussion

DDR pathways have been extensively studied across cancer types, and the success of PARPi in BRCA-associated cancers [19] has greatly advanced interest in these pathways for clinical exploitation as therapeutic targets as well as biomarkers of response to existing therapies [20,21]. Given the clinical significance of the DDR pathways, this is the first study aimed at elucidating genomic/transcriptomic alterations in 120 DDR genes and if the functional activity of the corresponding DDR pathways were associated with response to clinically approved chemotherapies for ovarian cancer.

Utilising a panel of cell lines with different histologic subtypes, we identified that HGSOC cell lines (Kuramochi, COV318, CAOV3, ES2, NIH-OVCAR3, UWB1.289, UWB1.289 + BRCA1, and COV362) were more sensitive to carboplatin, gemcitabine, topotecan and rucaparib, while the non-HGSOC cell lines (OAW42, A2780, CP70-B1, CP70-A2, IGROV1, and NUCOLL43) were more sensitive to doxorubicin and paclitaxel. Several earlier studies have attempted to analyse the genomic/transcriptomic/histological sub-type characterisation of ovarian cancer cell lines or correlate with response to chemotherapy [12,22,23]. However, one of the major differences from this study was the fact that the chemotherapy response was estimated by cellular dehydrogenase activity assay (MTT assay), which can be subject to variations in cell doubling time and effects of the drug on intermediary metabolism or unbalanced cell growth [24]. For the colony formation assay used here, the cells were incubated for varying times post drug treatment to accommodate the differences in proliferation rate and allowed to form colonies of at least 30 cells, ensuring at least five cell doublings post-drug exposure.

Genomic profiling of 120 DDR/PARP family genes identified frequent CNAs in genes involved in HRR and BER function and several PARP family proteins, which were exclusive to HGSOCs. PARP10 amplification and/or increased expression correlated with sensitivity to carboplatin and rucaparib, which was confirmed in the TCGA ovarian cancer dataset where it associated with platinum response. While the role of PARP10 in alleviating replication stress [25] has been well established, its likely role as a marker of platinum sensitivity is yet to be explored and demands further studies. PARP10 amplification co-occurred with RECQL4 amplification, both located on Chr 8q24.3, in both cell lines and ovarian cancers (TCGA). While RECQL4 has been associated with HRR function [26], its role in determining the HRR and NHEJ choice has also been well established [27]. However, the functional explanation for its association with platinum sensitivity needs investigation.

A strong positive correlation was observed between carboplatin and rucaparib sensitivity, which could be explained by genomic loss of BRCA gene/HRR dysfunction in both the cell line panel and patient ascites-derived primary cultures. NIH-OVCAR3, as we have shown previously [13], was an interesting cell line with high sensitivity to carboplatin and rucaparib despite a lack of functionally inactivating mutations in BRCA/other HRR genes but showing functional loss of HRR and high NHEJ activity. The critical role of NHEJ function in determining hypersensitivity of HRD cells to PARP inhibition was also previously identified by Patel et al. 2011 [28]. A similar trend was also observed for MMR deficient and proficient paired cell lines, where MMR-defective cell line CP70-A2 was significantly more sensitive to carboplatin, rucaparib, and topotecan but less sensitive to doxorubicin as compared to the MMR-proficient CP70-B1 cells. However, the increased sensitivity of MMR-defective cells was in contrast to available literature where MMR loss has been known to associate with platinum resistance [29]. This could be due to a likely impact on HRR function, which has been demonstrated in previous reports [30] and also observed here with a reduction in the RAD51-foci formation in MMR deficient CP70-A2 cells as compared to the MMR corrected CP70-B1 cells. Interestingly, the contrasting trends of response to carboplatin/rucaparib and doxorubicin were also seen in patient-derived primary cultures, with the HRD cancers being more sensitive to carboplatin and rucaparib but less sensitive to doxorubicin than the HRC cancers. In this study, we identified a positive correlation between survival following doxorubicin exposure and NHEJ functional activity, which correlated negatively with carboplatin and rucaparib. Lack of NHEJ function has been proposed to confer sensitivity to topoisomerase-II poisons like doxorubicin [31,32,33], and DNA-PKcs inhibitors have been reported to synergise with doxorubicin in their cytotoxic potential [34,35]. Hence, cancers with low NHEJ activity, which are likely to be resistant to carboplatin and rucaparib could be targetable by doxorubicin. Doxorubicin (topoisomerase II inhibitor) sensitivity also correlated negatively with topotecan (topoisomerase I inhibitor), confirming the concept of collateral sensitivity between topoisomerase I and II poisons [36].

Interestingly, intrinsic oxidative stress, which is an indirect indicator of baseline BER activity, measured in terms of 8-OHdG levels per µg of DNA, identified a negative correlation of survival with gemcitabine. Gemcitabine has a direct role in inducing oxidative stress by increasing ROS production [37]. These observations highlight that 8-OhdG could potentially be a marker for identifying cancers with reduced BER capacity, which may be more responsive to gemcitabine in second-line treatments. While 8-OHdG measurements can be done by simple immunohistochemical approaches, large scale clinical studies are needed to identify suitable cut-offs for the classification of HGSOCs as high or low with reliable sensitivity and specificity to predict response to gemcitabine.

## 5. Conclusions

In summary, our data highlights the importance of the genomic/transcriptomic as well as functional analysis of DDR pathways, which can guide the selection of chemotherapy choice for relapsed HGSOCs. As observed clinically, HRD cancer cell lines and primary cultures were more sensitive to carboplatin and rucaparib. Also, predictably cells with low NHEJ activity were more sensitive to doxorubicin, but interestingly, HRD cells were less sensitive to doxorubicin. Additionally, 8-OHdG levels, indicative of high intrinsic oxidative stress, was associated with increased sensitivity to gemcitabine. We also identified that common gene amplifications in ovarian cancers, like PARP10, associated with platinum response. This study, therefore, highlights the relevance of key DDR pathway alterations in determining chemotherapy response which demands further exploration in the clinic.

## 6. Patents

1. Helleday T and Curtin NJ. Therapeutic Compounds (PARP inhibitors in homologous repair/BRCA defective cancer) WO 2005/012305 A2
2. Boritzki TJ, Calvert AH, Curtin NJ, Dewji MR, Hostomsky Z, Jones C, Kaufman R, Klamerus KJ, Newell DR, Plummer ER, Reich SD, Steinfeldt HM, Stratford IJ, Thomas HR Williams KJ. Therapeutic Combinations Comprising PARP inhibitor WO/2006/033006
3. Falcon S, Reaper P, Pollard J, Curtin NJ, Middleton FK and Chen T. Method for measuring ATR inhibition mediated increases in DNA damage. WO2014055756A1

## Figures and Tables

**Table 1:** LC_50_ values (mean ± SD) and % survival at a given concentration (mean ± SD) of carboplatin, doxorubicin, gemcitabine, topotecan, paclitaxel, and rucaparib for the 14 cell line panel is listed below. Each data represents an average of three independent experiments. (Bold: Highest value; *Italics:* Lowest value).

Cell Line	Carboplatin		Doxorubicin		Gemcitabine		Topotecan		Paclitaxel		Rucaparib	
	LC_50_ (µM)	% Survival (10 µM)	LC_50_ (nM)	% Survival (100 nM)	LC_50_ (nM)	% Survival (30 nM)	LC_50_ (nM)	% Survival (30 nM)	LC_50_ (nM)	% Survival (30 nM)	LC_50_ (µM)	% Survival (10 µM)
Kuramochi	2.9 ± 0.6	6.2 ± 3.1	38.8 ± 3.3	17.6 ± 1.3	58.9 ± 18.9	56.4 ± 4.6	16.3 ± 1.5	29 ± 3.7	3.7 ± 1.5	**33.4 ± 3.3**	3 ± 0.5	26.3 ± 7.5
COV318	2.8 ± 0.2	20.5 ±2.6	35.3 ± 0.5	14.4 ± 0.8	17.8 ± 1.6	30.8 ± 0.7	17.5 ± 0.6	27.4 ± 1.5	**9.7 ± 0.2**	32.1 ± 4	2.8 ± 0.2	36.5 ± 2
CAOV3	3.1 ± 1.7	24.4 ± 3.6	*19.3 ± 0.7*	*0.8 ± 0.0*	*7 ± 0.2*	*7.1 ± 0.2*	16.1 ± 1.4	17 ± 2	*0.9 ± 0.1*	1.8 ± 0.9	2.4 ± 0.1	19.6 ± 2.3
ES2	17.3 ± 2.4	61.2 ± 4.3	**72.7 ± 5.2**	**35.3 ± 1.7**	13.6 ± 1.6	12.8 ± 3.2	13.6 ± 1.9	7.6 ±2.7	6.9 ± 0.2	1.3 ± 0.5	19.2 ± 0.9	71.1 ± 3.8
OAW42	2.7 ± 0.2	10.9 ± 0.9	59.2 ± 4.9	21.9 ± 1.7	**99.2 ± 8.9**	66.9 ± 5.2	12.7 ± 1.3	13.7 ± 2.1	2.6 ± 0.04	2.5 ± 0.8	3.7 ± 0.8	37.9 ± 2
A2780	3.5 ± 0.7	15.3 ± 4.3	45.3 ± 2.2	4 ± 1.5	26.1 ± 2.1	42.2 ± 4.6	17.5 ± 1.7	12 ± 5.4	2.3 ± 0.2	*0.8 ± 0.00*	4.4 ± 1.1	31.6 ± 2.6
CP70-B1	**22.1 ± 3.2**	**66.8 ± 4.5**	27.9 ± 0.4	1.1 ± 0.3	25.9 ± 1.2	39.3 ± 3.9	**42.9 ± 3.3**	**61 ± 3.6**	2.5 ± 0.1	1.5 ± 0.7	**>30**	**84.8 ± 2.6**
CP70-A2	7.3 ± 0.04	29.6 ± 1.6	52.1 ± 2.6	8 ± 0.4	25.2 ± 0.9	40.6 ± 1.6	11.8 ± 2	17.4 ± 1.7	2.9 ± 0.1	3.7 ± 1.4	10 ± 0.1	50.1 ± 0.1
IGROV1	2.3 ± 0.3	5.4 ± 1.7	42.7 ± 2.2	13.2 ± 0.7	8 ± 0.6	27.7 ± 4.2	12.5 ± 1.9	14.9 ± 3.1	2.5 ± 0.3	11.3 ± 1.1	8.9 ± 1.2	46.6 ± 3.7
UWB1.289 + BRCA1	1.3 ± 0.1	0.8 ± 0.00	62.3 ± 2.5	23.8 ± 1.6	22.2 ± 0.3	31.4 ± 2.1	10.5 ± 0.1	5.7 ± 1.04	2.5 ± 0.02	7.6 ± 0.6	2.8 ± 0.1	34.1 ± 2.9
NUCOLL43	7.8 ± 1	39.9 ± 4.2	43.5 ± 3	4.2 ± 0.3	71. ± 9.6	**69.4 ± 0.8**	15 ± 2.6	7.2 ± 2.2	2.5 ± 0.1	5.1 ± 1.3	20.8 ± 0.6	68.1 ± 1.6
NIH-OVCAR3	1.3 ± 0.3	0.8 ± 0.00	55 ± 3.4	16.9 ± 3.2	41.6 ± 2.1	58.8 ± 2	7.2 ± 0.6	11.6 ± 1.7	2.7 ± 0.1	16.1 ± 2.5	0.4 ± 0.06	0.8 ± 0.00
UWB1.289	*0.7 ± 0.03*	*0.8 ± 0.00*	44.9 ± 3.3	13.6 ± 0.7	8.2 ± 0.6	7.2 ± 0.8	*4.1 ± 0.6*	*0.08 ± 0.0*	2.6 ± 0.07	3.9 ± 1.2	*0.2 ± 0.02*	*0.8 ± 0.00*
COV362	2.6 ± 0.1	3.4 ± 0.5	28.3 ± 1.2	7.7 ± 0.2	20 ± 1	29.8 ± 2.1	12.9 ± 0.6	25.8 ± 2.5	2.7 ± 0.06	18.8 ± 1.3	*0.2± 0.01*	*0.8 ± 0.0*

## Data Availability

In house generated RNA-Seq data of cell lines generated using Illumina NextSeq with the HTG EdgeSeq Oncology biomarker panel assay is available at https://www.ncbi.nlm.nih.gov/geo/ (accessed on 18 October 2020) (GEO submission: GSE150942). Cancer Cell Line Encyclopedia (CCLE) and The Cancer Genome Atlas (TCGA) data were accessed from cbioportal (https://www.cbioportal.org/) (accessed on 12 January 2021).

## Supplementary Materials

The following are available online at https://www.mdpi.com/2072-6694/13/6/1420/s1, Figure S1: Distribution of LC50 values for (a) Carboplatin, (b) Doxorubicin, (c) Gemcitabine, (d) Topotecan, (e) Paclitaxel, and (f) Rucaparib, across the cell line panel. Figure S2: Distribution of survival % at a given concentration of (a) Carboplatin, (b) Doxorubicin, (c) Gemcitabine, (d) Topotecan, (e) Paclitaxel, and (f) Rucaparib, across the cell line panel. Figure S3: Hierarchical clustering of % Survival at fixed drug concentration for each of the14 cell lines in the panel. Figure S4: Correlation between in-house RNA-Seq data and published data on nine ovarian cancer cell lines to confirm the identity of the cell lines. Figure S5: Hierarchical clustering of Pearson’s correlation co-efficient of normalised gene expression values and % survival at a fixed concentration of the drug. Figure S6: Growth inhibition analysis of the patient ascites-derived primary cultures following treatment with carboplatin, rucaparib, doxorubicin, gemcitabine, topotecan, and paclitaxel. Figure S7: Correlation of functional activity of (A) the NHEJ pathway and (B) intrinsic oxidative stress with sensitivity to the different drugs. Table S1: Culture media used for the maintenance of the cell lines. Table S2: Cell line panel mutation or amplification status of frequent genomic alterations reported in High-Grade Serous Ovarian Cancers (cbioportal). Table S3: Correlation between % Survival at fixed drug concentrations and Area Under the Curve (AUC).Table S4: Homologous Recombination Repair defect (BRCA mutation and functional HRR status) and the chemosensitivity to six chemotherapy drugs for the patient ascites-derived primary cultures. Table S5: Pearson’s correlation analysis between %NHEJ activity and % survival at given concentrations of the drugs. Table S6: Pearson’s correlation analysis between Baseline 8-OHdG levels and % survival at given concentrations of the drugs

## References

[1] Bray F., Ferlay J., Soerjomataram I., Siegel R.L., Torre L.A., Jemal A. (2018). Global cancer statistics 2018: GLOBOCAN estimates of incidence and mortality worldwide for 36 cancers in 185 countries. CA A Cancer J. Clin..

[2] McCluggage W.G. (2011). Morphological subtypes of ovarian carcinoma: A review with emphasis on new developments and pathogenesis. Pathology.

[3] Colombo N., Sessa C., Bois A.D., Ledermann J., McCluggage W.G., McNeish I., Morice P., Pignata S., Ray-Coquard I., Vergote I. (2019). ESMO-ESGO consensus conference recommendations on ovarian cancer: Pathology and molecular biology, early and advanced stages, borderline tumours and recurrent disease. Ann. Oncol..

[4] Clamp A.R., James E.C., McNeish I.A., Dean A., Kim J.W., O’Donnell D.M., Hook J., Coyle C., Blagden S., Brenton J.D. (2019). Weekly dose-dense chemotherapy in first-line epithelial ovarian, fallopian tube, or primary peritoneal carcinoma treatment (ICON8): Primary progression free survival analysis results from a GCIG phase 3 randomised controlled trial. Lancet.

[5] Lee C.K., Scott C., Lindeman G.J., Hamilton A., Lieschke E., Gibbs E., Asher R., Badger H., Paterson R., Macnab L. (2019). Phase 1 trial of olaparib and oral cyclophosphamide in BRCA breast cancer, recurrent BRCA ovarian cancer, non-BRCA triple-negative breast cancer, and non-BRCA ovarian cancer. Br. J. Cancer.

[6] Gelmon K.A., Tischkowitz M., Mackay H., Swenerton K., Robidoux A., Tonkin K., Hirte H., Huntsman D., Clemons M., Gilks B. (2011). Olaparib in patients with recurrent high-grade serous or poorly differentiated ovarian carcinoma or triple-negative breast cancer: A phase 2, multicentre, open-label, non-randomised study. Lancet Oncol..

[7] Swisher E.M., Lin K.K., Oza A.M., Scott C.L., Giordano H., Sun J., Konecny G.E., Coleman R.L., Tinker A.V., O’Malley D.M. (2017). Rucaparib in relapsed, platinum-sensitive high-grade ovarian carcinoma (ARIEL2 Part 1): An international, multicentre, open-label, phase 2 trial. Lancet Oncol..

[8] Aghajanian C., Goff B., Nycum L.R., Wang Y.V., Husain A., Blank S.V. (2015). Final overall survival and safety analysis of OCEANS, a phase 3 trial of chemotherapy with or without bevacizumab in patients with platinum-sensitive recurrent ovarian cancer. Gynecol. Oncol..

[9] Bouberhan S., Pujade-Lauraine E., Cannistra S.A. (2019). Advances in the Management of Platinum-Sensitive Relapsed Ovarian Cancer. J. Clin. Oncol..

[10] Pennington K.P., Walsh T., Harrell M.I., Lee M.K., Pennil C.C., Rendi M.H., Thornton A., Norquist B.M., Casadei S., Nord A.S. (2014). Germline and somatic mutations in homologous recombination genes predict platinum response and survival in ovarian, fallopian tube, and peritoneal carcinomas. Clin. Cancer Res..

[11] Coleman R.L., Oza A.M., Lorusso D., Aghajanian C., Oaknin A., Dean A., Colombo N., Weberpals J.I., Clamp A., Scambia G. (2017). Rucaparib maintenance treatment for recurrent ovarian carcinoma after response to platinum therapy (ARIEL3): A randomised, double-blind, placebo-controlled, phase 3 trial. Lancet.

[12] Domcke S., Sinha R., Levine D.A., Sander C., Schultz N. (2013). Evaluating cell lines as tumour models by comparison of genomic profiles. Nat. Commun..

[13] Bradbury A., O’Donnell R., Drew Y., Curtin N.J., Sharma Saha S. (2020). Characterisation of Ovarian Cancer Cell Line NIH-OVCAR3 and Implications of Genomic, Transcriptomic, Proteomic and Functional DNA Damage Response Biomarkers for Therapeutic Targeting. Cancers.

[14] RL O.D., McCormick A., Mukhopadhyay A., Woodhouse L.C., Moat M., Grundy A., Dixon M., Kaufman A., Soohoo S., Elattar A. (2014). The use of ovarian cancer cells from patients undergoing surgery to generate primary cultures capable of undergoing functional analysis. PLoS ONE.

[15] Drew Y., Mulligan E.A., Vong W.T., Thomas H.D., Kahn S., Kyle S., Mukhopadhyay A., Los G., Hostomsky Z., Plummer E.R. (2011). Therapeutic potential of poly(ADP-ribose) polymerase inhibitor AG014699 in human cancers with mutated or methylated BRCA1 or BRCA2. J. Natl. Cancer Inst..

[16] Bennardo N., Cheng A., Huang N., Stark J.M. (2008). Alternative-NHEJ is a mechanistically distinct pathway of mammalian chromosome break repair. PLoS Genet..

[17] (2011). Integrated genomic analyses of ovarian carcinoma. Nature.

[18] Mukhopadhyay A., Plummer E.R., Elattar A., Soohoo S., Uzir B., Quinn J.E., McCluggage W.G., Maxwell P., Aneke H., Curtin N.J. (2012). Clinicopathological features of homologous recombination-deficient epithelial ovarian cancers: Sensitivity to PARP inhibitors, platinum, and survival. Cancer Res..

[19] Jonsson P., Bandlamudi C., Cheng M.L., Srinivasan P., Chavan S.S., Friedman N.D., Rosen E.Y., Richards A.L., Bouvier N., Selcuklu S.D. (2019). Tumour lineage shapes BRCA-mediated phenotypes. Nature.

[20] Hosoya N., Miyagawa K. (2014). Targeting DNA damage response in cancer therapy. Cancer Sci..

[21] O’Connor M.J. (2015). Targeting the DNA Damage Response in Cancer. Mol. Cell.

[22] Anglesio M.S., Wiegand K.C., Melnyk N., Chow C., Salamanca C., Prentice L.M., Senz J., Yang W., Spillman M.A., Cochrane D.R. (2013). Type-specific cell line models for type-specific ovarian cancer research. PLoS ONE.

[23] Beaufort C.M., Helmijr J.C., Piskorz A.M., Hoogstraat M., Ruigrok-Ritstier K., Besselink N., Murtaza M., van Ĳcken W.F., Heine A.A., Smid M. (2014). Ovarian cancer cell line panel (OCCP): Clinical importance of in vitro morphological subtypes. PLoS ONE.

[24] Haibe-Kains B., El-Hachem N., Birkbak N.J., Jin A.C., Beck A.H., Aerts H.J., Quackenbush J. (2013). Inconsistency in large pharmacogenomic studies. Nature.

[25] Schleicher E.M., Galvan A.M., Imamura-Kawasawa Y., Moldovan G.L., Nicolae C.M. (2018). PARP10 promotes cellular proliferation and tumorigenesis by alleviating replication stress. Nucleic Acids Res..

[26] Lu H., Shamanna R.A., Keijzers G., Anand R., Rasmussen L.J., Cejka P., Croteau D.L., Bohr V.A. (2016). RECQL4 Promotes DNA End Resection in Repair of DNA Double-Strand Breaks. Cell Rep..

[27] Lu H., Shamanna R.A., de Freitas J.K., Okur M., Khadka P., Kulikowicz T., Holland P.P., Tian J., Croteau D.L., Davis A.J. (2017). Cell cycle-dependent phosphorylation regulates RECQL4 pathway choice and ubiquitination in DNA double-strand break repair. Nat. Commun..

[28] Patel A.G., Sarkaria J.N., Kaufmann S.H. (2011). Nonhomologous end joining drives poly(ADP-ribose) polymerase (PARP) inhibitor lethality in homologous recombination-deficient cells. Proc. Natl. Acad. Sci. USA.

[29] Martin L.P., Hamilton T.C., Schilder R.J. (2008). Platinum resistance: The role of DNA repair pathways. Clin. Cancer Res..

[30] Mohindra A., Hays L.E., Phillips E.N., Preston B.D., Helleday T., Meuth M. (2002). Defects in homologous recombination repair in mismatch-repair-deficient tumour cell lines. Hum. Mol. Genet..

[31] Caldecott K., Banks G., Jeggo P. (1990). DNA double-strand break repair pathways and cellular tolerance to inhibitors of topoisomerase II. Cancer Res..

[32] Jeggo P.A., Caldecott K., Pidsley S., Banks G.R. (1989). Sensitivity of Chinese hamster ovary mutants defective in DNA double strand break repair to topoisomerase II inhibitors. Cancer Res..

[33] Munck J.M., Batey M.A., Zhao Y., Jenkins H., Richardson C.J., Cano C., Tavecchio M., Barbeau J., Bardos J., Cornell L. (2012). Chemosensitization of cancer cells by KU-0060648, a dual inhibitor of DNA-PK and PI-3K. Mol. Cancer Ther..

[34] Fok J.H.L., Ramos-Montoya A., Vazquez-Chantada M., Wijnhoven P.W.G., Follia V., James N., Farrington P.M., Karmokar A., Willis S.E., Cairns J. (2019). AZD7648 is a potent and selective DNA-PK inhibitor that enhances radiation, chemotherapy and olaparib activity. Nat. Commun..

[35] Tavecchio M., Munck J.M., Cano C., Newell D.R., Curtin N.J. (2012). Further characterisation of the cellular activity of the DNA-PK inhibitor, NU7441, reveals potential cross-talk with homologous recombination. Cancer Chemother. Pharmacol..

[36] Parchment R.E., Pessina A. (1998). Topoisomerase I inhibitors and drug resistance. Cytotechnology.

[37] Ju H.Q., Gocho T., Aguilar M., Wu M., Zhuang Z.N., Fu J., Yanaga K., Huang P., Chiao P.J. (2015). Mechanisms of Overcoming Intrinsic Resistance to Gemcitabine in Pancreatic Ductal Adenocarcinoma through the Redox Modulation. Mol. Cancer Ther..

