# Supplementary material for: Genomic, Transcriptomic, and Functional Alterations in DNA Damage Response Pathways as Putative Biomarkers of Chemotherapy Response in Ovarian Cancer
Source: Cancers (Basel). 2021 Mar 20;13(6):1420. doi: 10.3390/cancers13061420 (PMC8003626; doi:10.3390/cancers13061420)
Supplement: Supplementary file 1 [file cancers-13-01420-s001.pdf]

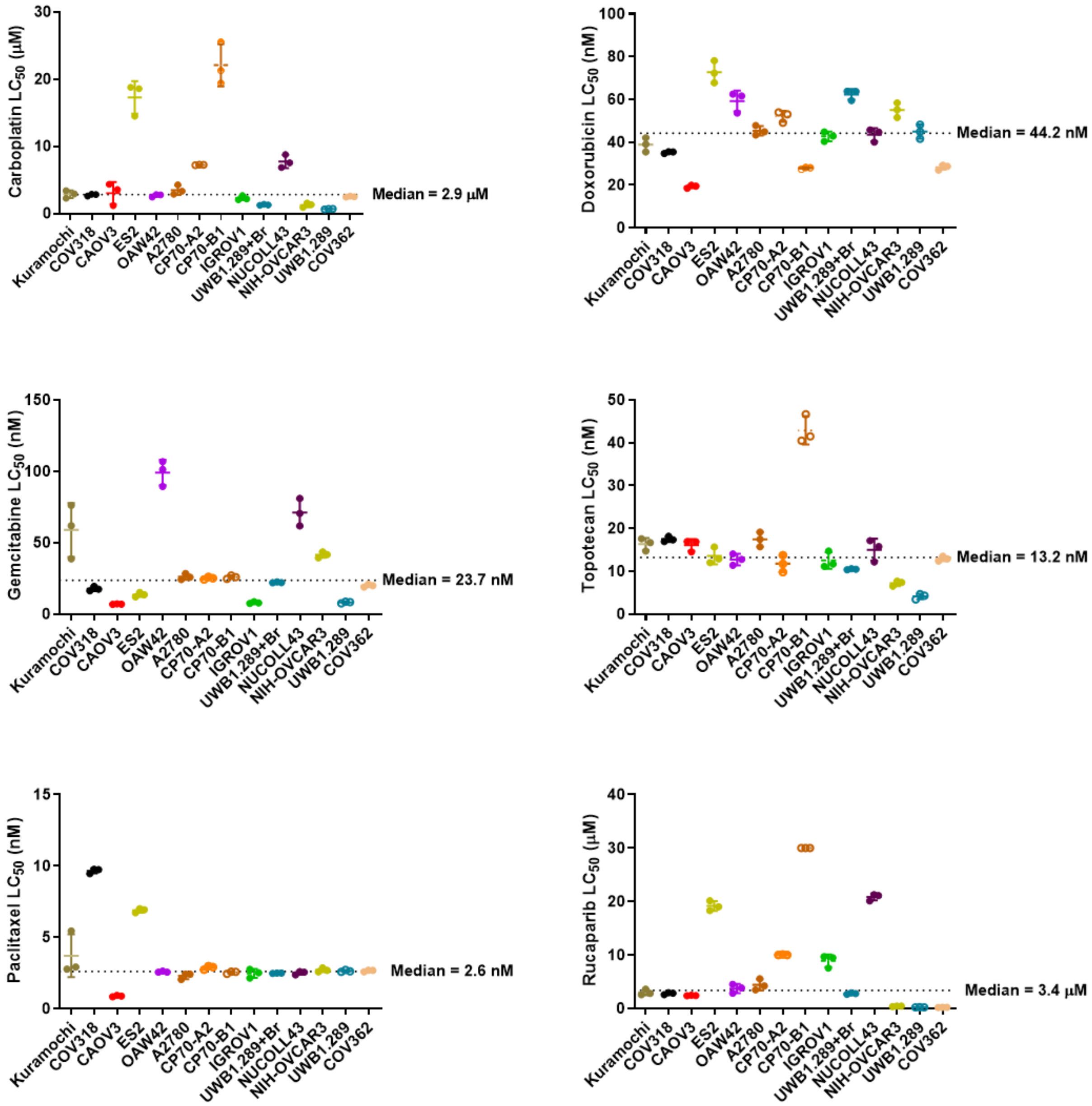

Supplementary Figure S1: Distribution of LC_{50} values for (a) Carboplatin, (b) Doxorubicin, (c) Gemcitabine, (d) Topotecan, (e) Paclitaxel, and (f) Rucaparib, across the cell line panel. The dotted line represents the median LC_{50} value. Each data point represents Mean ± SD LC_{50} for that cell line.

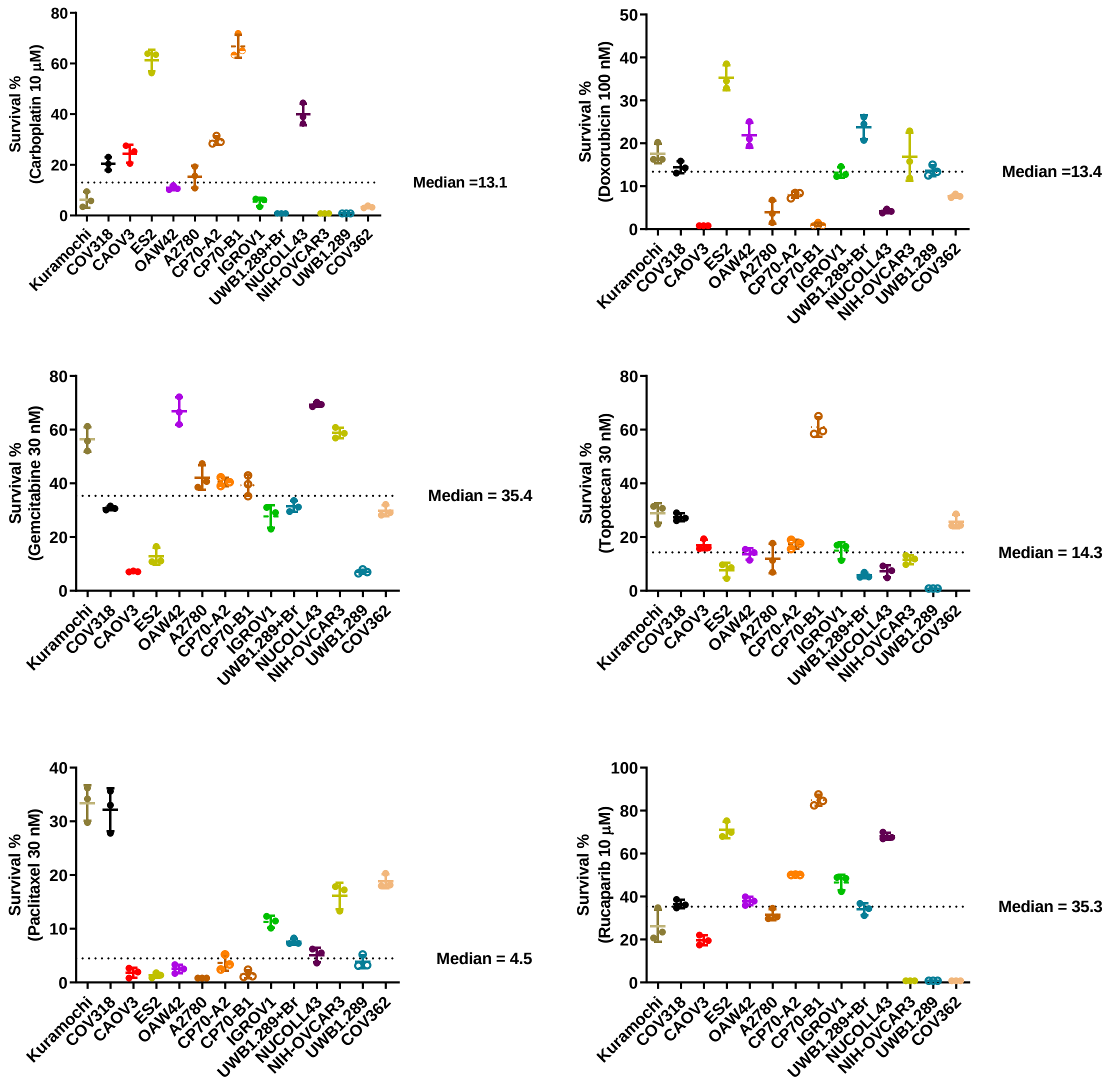

Supplementary Figure 2: Distribution of survival % at given concentration of (a) Carboplatin, (b) Doxorubicin, (c) Gemcitabine, (d) Topotecan, (e) Paclitaxel, and (f) Rucaparib, across the cell line panel. The dotted line represents the median survival % value of the cell lines. Each data represents Mean ± SEM of the survival % of 3 independent experiments for that cell line.

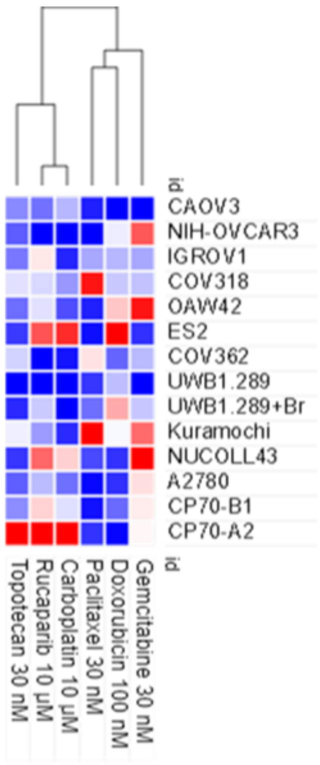

% Survival

Supplementary Figure S3: Hierarchical clustering of % Survival at fixed drug concentration for each of the14 cell lines in the panel.

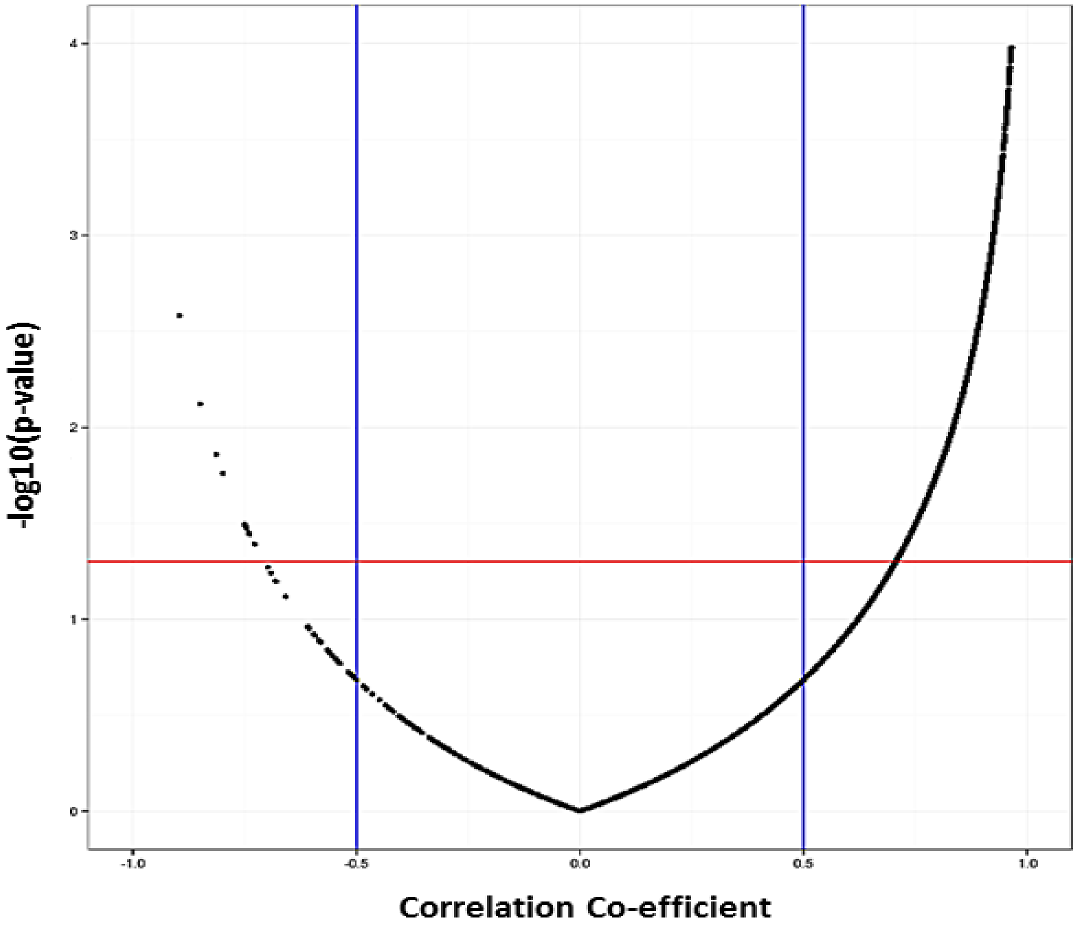

Supplementary Figure S4: Correlation between in-house RNA-Seq data and published data on 9 ovarian cancer cell lines to confirm the identity of the cell lines. Pearson's correlation coefficients and the corresponding p-values between the two datasets for 2495 genes used to generate the volcano plot shows a positive correlation for most genes. The average Pearson's correlation coefficient across all genes was 0.58. [The red line indicates p-value of 0.05 and the blue line indicates an r-value of 0.5].

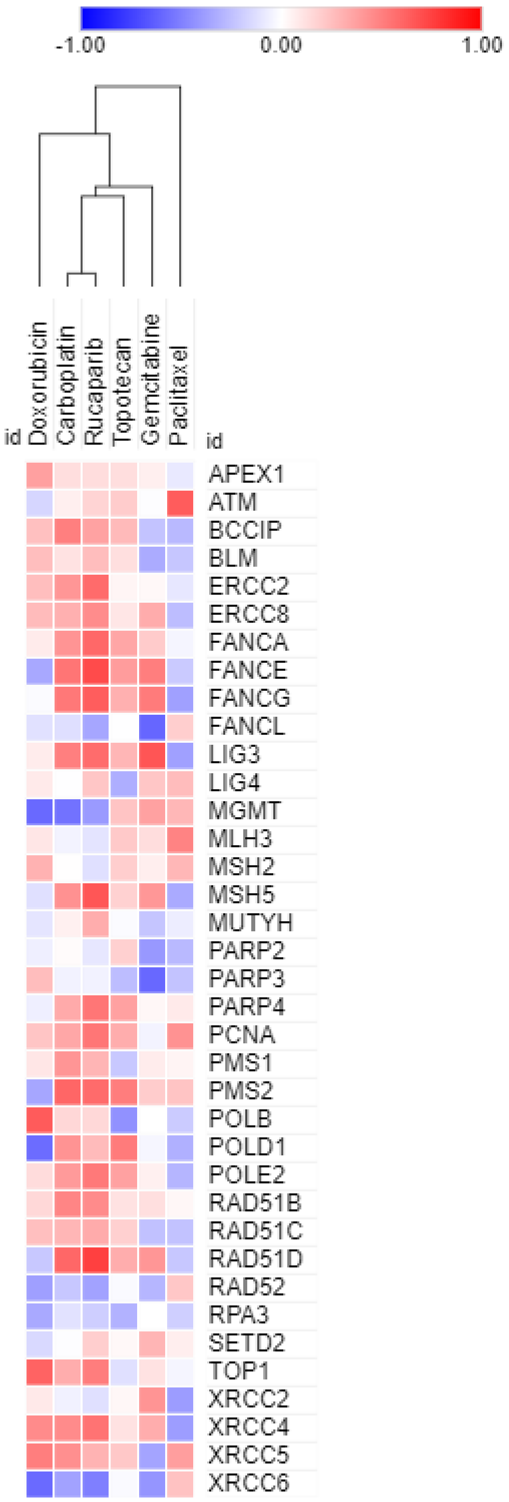

Supplementary Figure S5: Hierarchical clustering of Pearson's correlation co-efficient of normalized gene expression values and % survival at a fixed concentration of drug (Carboplatin: 10 µM; Doxorubicin: 100 nM; Gemcitabine: 30 nM; Topotecan: 30 nM; Paclitaxel: 30 nM and Rucaparib: 10 µM)

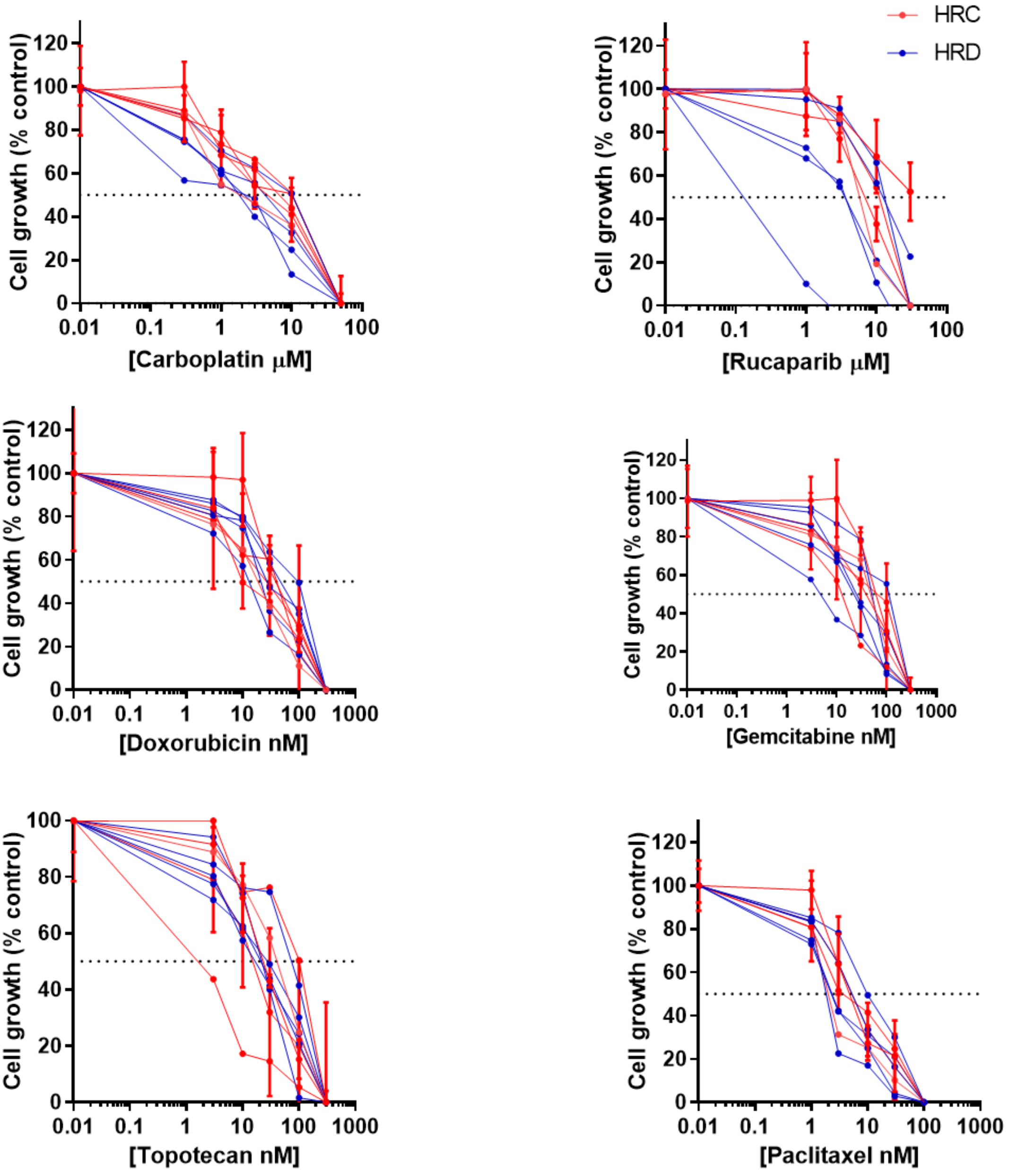

Supplementary Figure S6: Growth inhibition analysis of the patient ascites-derived primary cultures following treatment with carboplatin, rucaparib, doxorubicin, gemcitabine, topotecan and paclitaxel. Dotted line represents the concentration corresponding to the GI_{50} value for each sample and the corresponding drug. The samples are classified into HRR competent (HRC, red lines) and HRR defective (HRD, blue lines) identified using functional γH2AX-RAD51 foci formation assay (data not shown).

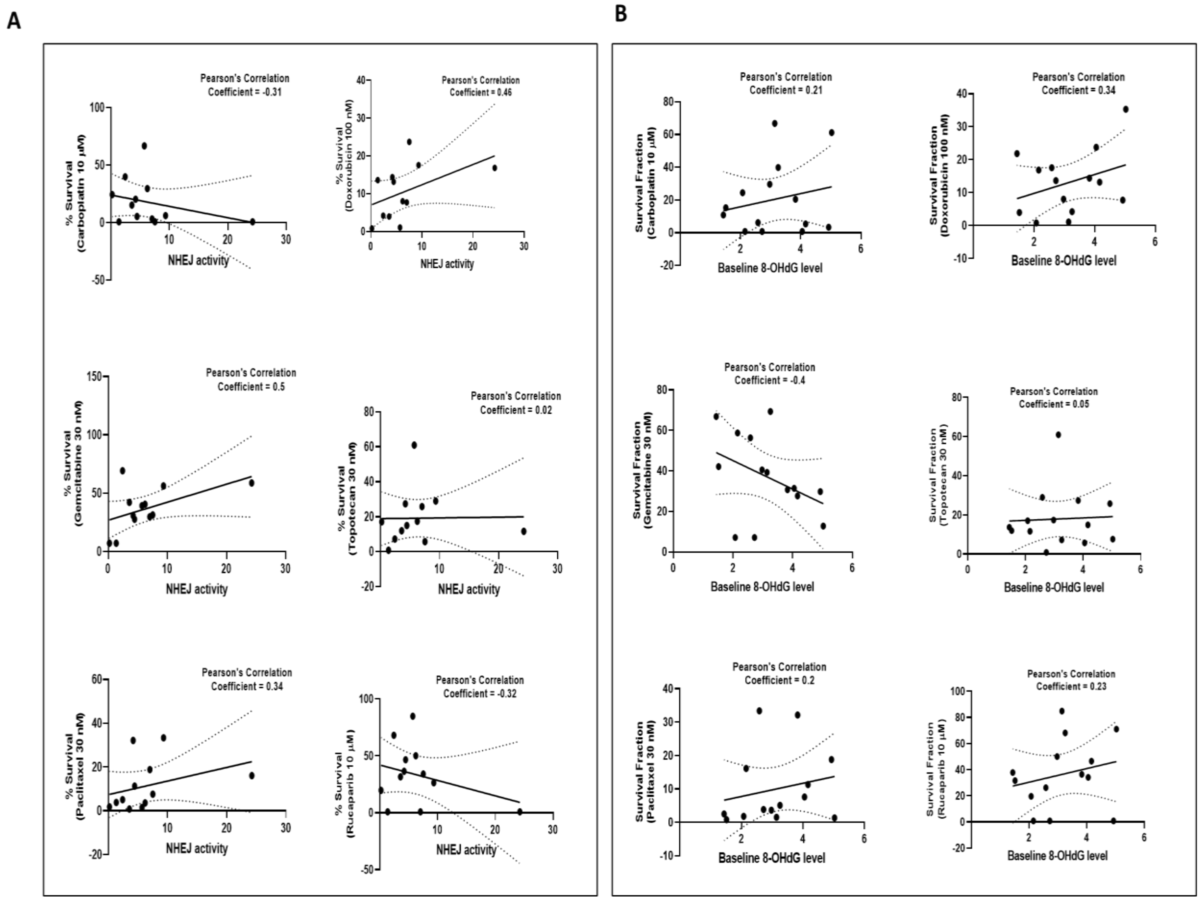

Supplementary Figure S7: Correlation of functional activity of (A) NHEJ pathway and (B) intrinsic oxidative stress with sensitivity to the different drugs

Table S1: Culture media used for the maintenance of the cell lines

Cell Line	Original Histopathological Classification	Ranking as HGSOC/non-HGSOC	Morphology	Tumour Source	Pre-culture clinical treatment status	Culture Media	Doubling Time (Hrs)
Kuramochi	Undifferentiated adenocarcinoma	HGSOC	Epithelial	Peritoneal ascites	Unknown	RPMI 1640, 10% FBS	40
COV318	HGS	HGSOC	Epithelial	Peritoneal ascites	Unknown	DMEM, 10% FBS	67
CAOV3	Adenocarcinoma	HGSOC	Epithelial	Ovarian solid tumour	Unknown	DMEM, 10% FBS	73
ES2	Clear Cell	HGSOC	Spindle	Ovarian solid tumour	Unknown [The cells exhibit low to moderate resistance to a number of chemotherapeutic agents including doxorubicin, cisplatin, carmustine, etoposide and cyanomorpholinodoxorubicin (MRA-CN)]	RPMI 1640, 10% FBS	21
OAW42	Cystadenocarcinoma	Non-HGSOC	Epithelial	Peritoneal ascites	At passage 4 the cell line showed resistance to doxorubicin [adriamycin (ADM)], phosphoramide mustard (PM), and cisplatin [cis-dichlorodiammineplatinum(II)] (CIS) but rapidly reverted to CIS sensitivity. At passage 25 the cell line was still resistant to ADM and PM (Wilson et al, 1984)	RPMI 1640, 10% FBS	49
A2780	Adenocarcinoma	Non-HGSOC	Round	Ovarian solid tumour	Untreated	RPMI 1640, 10 % FBS	21
CP70-B1	N/A	Non-HGSOC	Round	Derivative of CP70 which are derivatives of A2780	Derivative of CP70 which are derivatives of A2780	RPMI 1640, 10 % FBS + 200 µg/ml Hygromycin B	27
CP70-A2	N/A	Non-HGSOC	Round	Derivative of CP70 which are derivatives of A2780	Derivative of CP70 which are derivatives of A2780	RPMI 1640, 10 % FBS + 200 µg/ml Hygromycin B	20

IGROV1	Mixed endometrioid, serous, cell cell, undifferentiated	Non-HGSOC	Epithelial	Ovarian solid tumour	Untreated	RPMI 1640, 10 % FBS	33
UWB1.289+BRCA1	HGS	HGSOC	Epithelial	Recurrent Ovarian Cancer (earlier Breast Cancer)	Treated	50% RPMI-1640 + 50% MEGM Bullet Kit medium + 200 µg/ml G-418	51
NUCOLL43	Clear Cell	Non-HGSOC	Epithelial	Peritoneal acsites	Untreated	RPMI 1640, 20 % FBS	54
NIH-OVCAR3	Adenocarcinoma	HGSOC	Epithelial	Peritoneal acsites	Combination chemotherapy with Cyclophosphamide, Adriamycin, and cisplatin	RPMI 1640, 10 % FBS	53
UWB1.289	Serous	Non-HGSOC	Epithelial	Recurrent Ovarian Cancer (earlier Breast Cancer)	Treated	50% RPMI-1640 + 50% MEGM Bullet Kit medium	52
COV362	Endometriod	HGSOC	Spindle	Pleural effusion	Unknown	DMEM, 10% FBS	73

HGSOC: High Grade Serous Ovarian Cancer; FBS: Fetal Bovine Serum

Table S2: Cell line panel mutation or amplification status of frequent genomic alterations reported in High Grade Serous Ovarian Cancers (cbioportal)

**Cell line**	**TP53**	**BRCA 1**	**BRCA 2**	**CCNE 1**	**RB1**	**MMR**	**MYC**	**ARID 1A**	**EMS Y**
**Kuramochi**	Mut		Mut				Amp		
**COV318**	Mut	Low Exp		Amp					
**CAOV3**	Mut		Amp		Low Exp				
**ES2**	Mut								
**OAW42**	WT	Mut			High Exp		Amp	Mut	
**A2780**	WT				High Exp	Comp		Mut	
**CP70-B1**	Mut					Comp			
**CP70-A2**	Mut					Def			
**IGROV1**	Mut	Mut	Mut				Mut		
**UWB1.289+BRCA1**	Mut	WT							
**NUCOLL43**	Null								
**NIH-OVCAR3**	Mut			Amp					Amp
**UWB1.289**	Mut	Mut							
**COV36**	Mut	Mut			Low Exp		Amp		Amp

Mut: Mutated; WT: Wild-type; Amp: Amplification; Low Exp: Low mRNA Expression; High Exp: High mRNA expression; Comp: Competent; Def: Defective

Table S3: Correlation between % Survival at fixed drug concentration and Area Under the Curve (AUC) of the survival curves of the 14 cell lines for each drug

	**Carboplatin**	**Doxorubicin**	**Gemcitabine**	**Topotecan**	**Paclitaxel**	**Rucaparib**
**Pearson's Correlation coefficient**	0.992393	0.95823	0.858857	0.763185	0.951332	0.993379
**p-val**	<0.0001	<0.0001	<0.0001	0.0015	<0.0001	<0.0001

Table S4: Homologous Recombination Repair defect (BRCA mutation and functional HRR status) and the chemosensitivity to six chemotherapy drugs for the patient ascites-derived primary cultures

**Sample List**	**BRCA status**	**HRR Status**	**Carboplatin GI50 (μM)**	**Rucaparib GI50 (μM)**	**Doxorubicin GI50 (nM)**	**Gemcitabine GI50 (nM)**	**Paclitaxel GI50 (nM)**	**Topotecan GI50 (nM)**
NEOCATS-A-003	gBRCA wildtype	HRD	2.4	4	97.6	24.4	2.1	23.5
NEOCATS-A-005	gBRCA wildtype	HRC	10.5	>30	27.6	44.6	6.2	17.4
NEOCATS-A-014	gBRCA wildtype	HRD	2.5	4	55.6	60.6	2.5	24.1
NEOCATS-A-017	Unknown	HRD	10.6	14	23.5	26.5	6.2	19.2
NEOCATS-A-018	gBRCA wildtype	HRC	7.7	>30	10	14.2	2.7	4.1
NEOCATS-A-022	Unknown	HRD	4.9	0.56	14.7	5.6	2.5	28.5
NEOCATS-A-023	gBRCA1 mutant	HRC	2.1	7	21.3	57.1	2.2	47.5
NEOCATS-A-026	gBRCA wildtype	HRC	5.4	7.8	44.2	71.3	5.6	25.3
NEOCATS-A-029	Unknown	HRC	10.6	11.7	48.7	75.2	4.1	101.7
NEOCATS-A-030	somatic BRCA mutant	HRD	2.1	14.8	28.1	119.6	9.9	82.1

Table S5: Pearson's correlation analysis between %NHEJ activity and % survival at given concentrations of the drugs

**Drug**	**Correlation coefficient**	**Outliers (ES2 and OAW42) removed**
Carboplatin 10 μM	-0.31	-0.31
Doxorubicin 100 nM	0.11	0.46
Gemcitabine 30 nM	0.36	0.5
Topotecan 30 nM	0.08	0.02
Paclitaxel 30 nM	0.4	0.34
Rucaparib 10 μM	-0.35	-0.32

Table S6: Pearson's correlation analysis between Baseline 8-OHdG levels and % survival at given concentrations of the drugs

**Drug**	**Correlation co-efficient**
Carboplatin 10 μM	0.21
Doxorubicin 100 nM	0.34
Gemcitabine 30 nM	-0.4
Topotecan 30 nM	0.05
Paclitaxel 30 nM	0.2
Rucaparib 10 μM	0.23
